# Improving prostate MRI quality assessment across diverse evaluators and institutional practice through a PI-QUAL v2 lecture: a pilot study

**DOI:** 10.1186/s13244-026-02450-x

**Published:** 2026-10-29

**Authors:** Lili Xu, Xu Wang, Yitao Liu, Kai Li, Xiaoxiao Zhang, Hailong Zhou, Gumuyang Zhang, Jianfeng Yang, Hongxia Ni, Liming Yang, Yifeng Zheng, Dong Liu, Guoqing Xiao, Zufeng Ge, Tianlu Feng, Yu Feng, Clare Allen, Caroline M. Moore, Francesco Giganti, Hao Sun, Lei Shi

**Affiliations:** 1 Department of Radiology, Zhejiang Cancer Hospital, Hangzhou Institute of Medicine (HIM), Chinese Academy of Sciences, Hangzhou, China; 2 Department of Radiology, State Key Laboratory of Complex Severe and Rare Disease, Peking Union Medical College Hospital, Peking Union Medical College, Chinese Academy of Medical Sciences, Beijing, China; 3 Department of Radiology, Shaoxing People’s Hospital, Shaoxing, China; 4 Department of Radiology, Huzhou Central Hospital, Huzhou, China; 5 Department of Radiology, Fenghua People’s Hospital, Ningbo, China; 6 Department of Radiology, University College London Hospital NHS Foundation Trust, London, UK; 7 Division of Surgery and Interventional Science, University College London, London, UK; 8 Department of Urology, University College London Hospital NHS Foundation Trust, London, UK

**Keywords:** Prostate, Magnetic resonance imaging, Prostate imaging quality, Quality control

## Abstract

**Objective:**

To evaluate the impact of a prostate imaging quality (PI-QUAL) v2 lecture on the accuracy and inter-reader agreement of prostate MRI image-quality assessment among radiologists and technologists from diverse professional backgrounds and institutions, and to evaluate its role in institutional-level quality improvement through a quality control (QC) process.

**Materials and methods:**

Ten participants (five radiologists and five technologists) from five different hospitals independently evaluated 15 prostate MRIs before and after a standardized PI-QUAL v2 lecture. Reference scores were established by two experienced radiologists. Concordance with reference was assessed using accuracy and balanced accuracy. Inter-reader agreement was measured using percent agreement (PA) and Gwet’s AC1. Subgroup analyses were conducted based on participant characteristics. For the quality assurance and QC (QA/QC), each center submitted 15 prostate MRIs before and after the lecture. Image quality was evaluated centrally to assess institutional improvement.

**Results:**

Overall accuracy increased from 66.7% to 76.7%, and balanced accuracy increased from 62.9% to 84.2%; the mixed-effects model indicated significantly higher post-lecture accuracy (*p* = 0.038). Accuracy was also higher across all subgroups. Inter-reader agreement improved from PA 57.6% to 72.6% and Gwet’s AC1 from 0.214 to 0.466. Through the QA/QC process, three centers initially failed to meet PI-QUAL v2 technical criteria. After feedback, all post-intervention scans achieved PI-QUAL v2 scores > 1.

**Conclusion:**

A structured PI-QUAL v2 lecture improved both the accuracy and inter-reader agreement of prostate MRI image-quality assessments among diverse evaluators. Incorporating QA/QC further enhanced institutional image quality. PI-QUAL v2 training is a feasible strategy for standardizing prostate MRI quality.

**Key Points:**

***Question*** Does a structured PI-QUAL v2 lecture for radiologists and technologists improve prostate MRI quality assessment performance and institutional imaging practice across institutions?

***Findings*** PI-QUAL v2 training improved accuracy (66.7% to 76.7%) and inter-reader agreement in prostate MRI quality assessment, while QA/QC feedback enhanced institutional imaging quality.

***Clinical relevance statement*** Structured PI-QUAL v2 education combined with quality assurance and quality control feedback improves evaluator consistency and institutional prostate MRI quality, facilitating standardized image-quality assessment and broader implementation of high-quality prostate MRI in clinical radiology.

**Graphical Abstract:**

**Figure:**
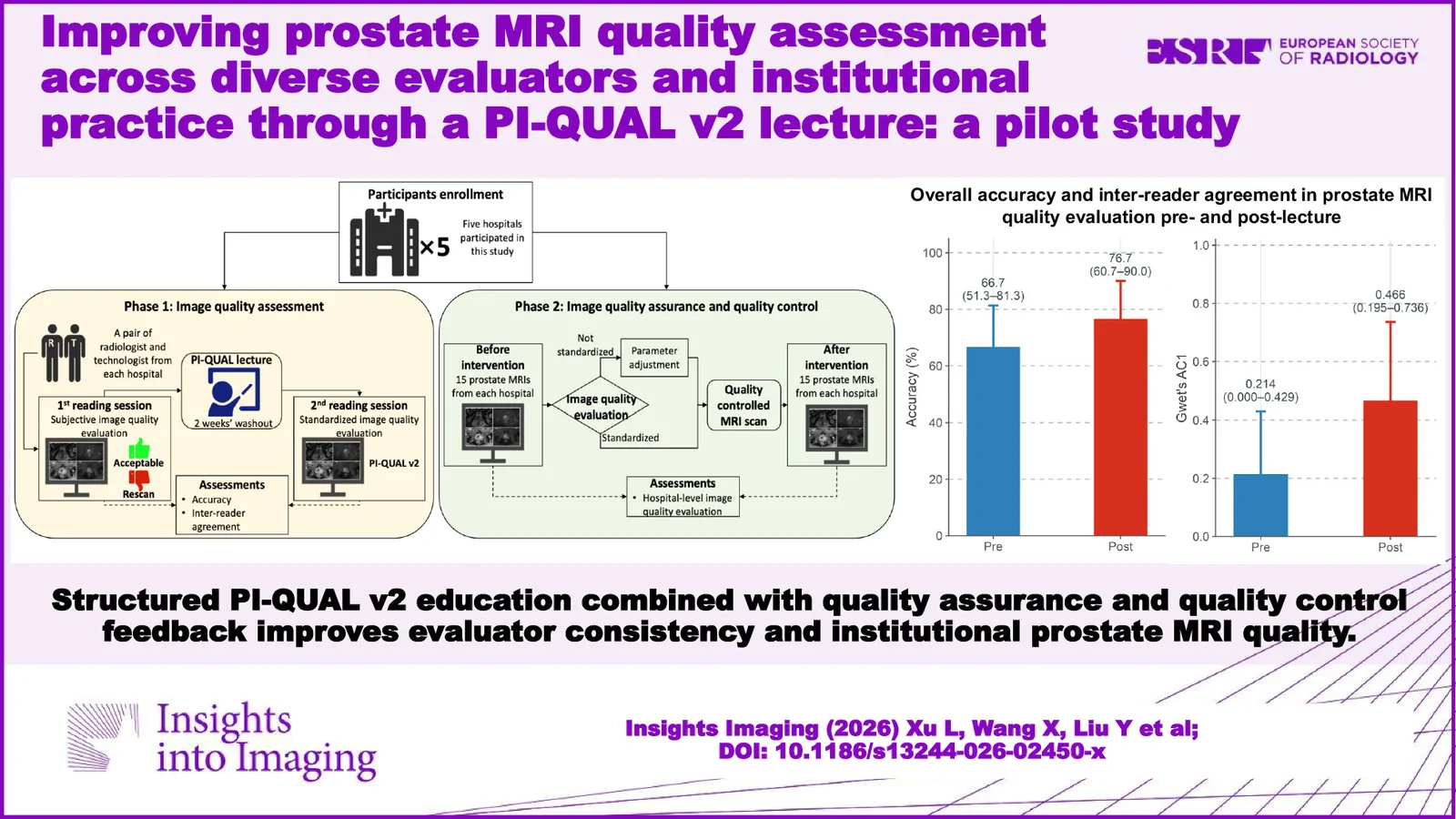

## Introduction

Prostate MRI plays an essential role in the detection, treatment decision-making, and diagnosis of relapse after the treatment of prostate cancer [1]. To standardize the acquisition and interpretation of prostate MRI, the Prostate Imaging Reporting and Data System (PI-RADS) was first introduced in 2012 [2], and has since evolved to the current version (2.1), released in 2019 [3]. The latest version outlines minimal technical requirements for standardized prostate MRI, including sequence-specific slice thickness, the inclusion of high b-value diffusion-weighted imaging (DWI) (b ≥ 1400 s/mm²), and appropriate temporal resolution (≤ 15 s) for dynamic contrast-enhanced (DCE) imaging [3].

Despite these standardized guidelines, compliance with PI-RADS v2.1 remains variable across institutions [4–7]. This variability is likely due to differences in scanner capabilities and protocol implementation, ultimately leading to inconsistencies in image quality. In a recent international quality assurance study of prostate MRI (GLIMPSE study) from the researchers of the PRIME study, involving 71 MRI scanners across 41 centers in 18 countries, 68% (48/71) of the scanners failed to meet the technical parameters defined by PI-RADS v2.1 and required protocol improvements [8].

Non-standardized acquisition can result in suboptimal image quality, thereby impairing diagnostic performance [9]. Prior to 2020, the criteria used to assess prostate MRI quality varied considerably among studies. In response to this, the prostate imaging quality (PI-QUAL) scoring system was introduced in order to provide a standardized and quantifiable method for evaluating image quality [10]. PI-QUAL was updated to version 2 in 2024, addressing some of the limitations of the original version and enabling its use in both multiparametric and biparametric (i.e., without intravenous contrast medium) MRI protocols [11]. The updated version simplifies assessment by reducing the number of evaluated criteria from 34 to 10 and introducing a more practical 3-point scoring system, combining technical and visual assessments of image quality.

While PI-QUAL offers a unified framework for evaluating image quality, inter-reader agreement remains variable and is often influenced by the evaluator’s level of experience [12, 13]. To mitigate this, the results from a dedicated PI-QUAL training curriculum demonstrated significant improvements in image-quality assessment using PI-QUAL v1 among participants from multidisciplinary backgrounds, including radiologists, urologists, physicists, and computer scientists [14]. Nevertheless, it remains unclear how such training, when delivered to key partners in prostate MRI (i.e., radiologists and technologists from the same institution), might affect their understanding of prostate MRI quality and influence image quality in their clinical scanning practice.

To address this gap, we conducted a pilot study to assess the impact of an online structured PI-QUAL lecture on reader performance and institutional image quality. The study consisted of two phases. Phase I aimed to assess whether the lecture could improve image-quality assessment and inter-observer agreement among readers with diverse backgrounds. Phase II aimed to evaluate whether the lecture and its associated quality assurance recommendations could lead to improvements in institutional MRI quality over time.

## Materials and methods

### Participant enrollment

This prospective, multicenter pilot study was approved by the Institutional Review Board (IRB No. IRB-2025-683 [IIT]) and was part of a clinical research Bracco fellowship under the umbrella of the European School of Radiology. Participating hospitals were required to meet the following inclusion criteria: (1) routinely perform biparametric or multiparametric prostate MRI; (2) conduct more than 15 prostate MRI examinations per month; (3) agree to receive image-quality improvement recommendations from the lecture team and to undergo post-lecture quality assessment.

A total of five hospitals were enrolled. From each hospital, one radiologist and one technologist who were actively involved in prostate MRI interpretation or scanning were invited to attend the online PI-QUAL v2 lecture. All participants were required to have basic knowledge of PI-RADS v2.1 and use it in clinical practice.

Radiologists were classified by experience according to the number of prostate MRI diagnoses [15]: Expert: > 1000 cases; Intermediate: 400–1000 cases; Beginner: ≤ 400 cases. As there is no standardized definition of technologist expertise, a similar volume-based classification was adopted using the number of prostate MRI scans performed.

### MR examinations for evaluation

Fifteen prostate MRI cases were purposefully selected by the study coordinator (L.X.) from two institutions (ZJCH and PUMCH) and three MRI scanners (GE DISCOVERY 750, Philips Ingenia Elition X, and Philips Ingenia CX) to achieve a balanced representation of PI-QUAL categories, institutions, and scanner platforms, thereby ensuring adequate representation of different image quality levels and acquisition settings. All cases were from patients with elevated PSA and an unknown history of prostate cancer. The images were anonymized and distributed to participants for evaluation. Detailed case information is provided in Table S1.

### Phase I—Image-quality assessment before and after PI-QUAL lecture

Phase I aimed to evaluate whether a PI-QUAL v2 lecture could improve participants’ accuracy and consistency in prostate MRI quality assessment.

Before the lecture, participants independently assessed the 15 anonymized cases and submitted their answers via an online platform (https://www.wjx.cn/). They were instructed to evaluate image quality based solely on personal experience and to complete a baseline survey including information on their professional background. The pre-lecture survey template is shown in Table S2. In this survey, participants were required to provide their hospital affiliation, their role (radiologist or technologist), professional title (junior, intermediate, or senior), experience in prostate MRI (beginner, intermediate, or expert), and knowledge of PI-QUAL v2 (none, basic, or full).

Following the baseline assessment, participants attended a live, 1.5-h online lecture on prostate MRI quality delivered via Tencent Meeting on May 8, 2025. The session was led by a prostate MRI expert (L.X., with experience reporting over 1500 prostate MRI scans), supervised by another expert (F.G., with > 2500 prostate scans annually), who is also a co-developer of the PI-QUAL system.

The lecture included three modules: (1) Fundamentals: Clinical importance of prostate MRI, PI-RADS scanning parameters, and the impact of image quality on diagnostic performance; (2) PI-QUAL overview: Evolution of the PI-QUAL system, v2 scoring structure, technical and subjective criteria; (3) Practical examples: Visual references for each PI-QUAL criterion to facilitate understanding and standardization.

**Fig. 1:**
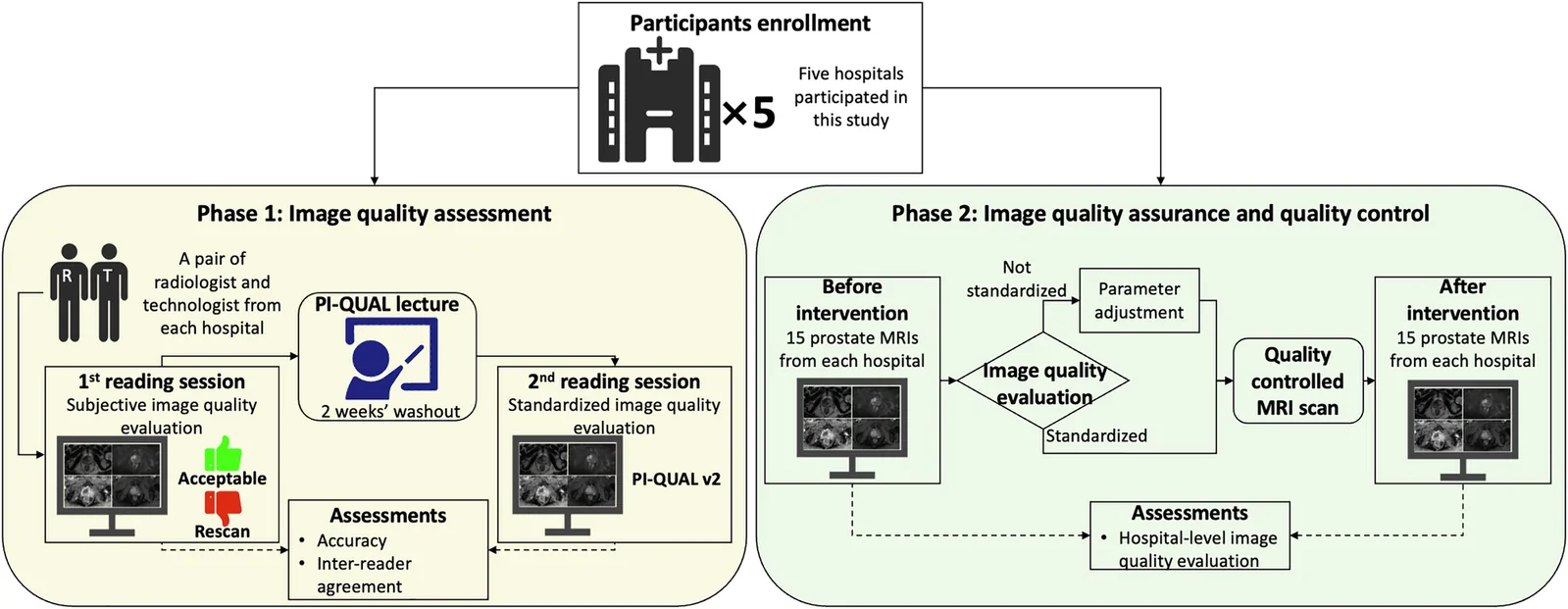
Research flowchart. This multicenter study involved five hospitals, each contributing one radiologist and one technologist actively engaged in prostate MRI. In Phase I, participants independently assessed prostate MRI quality before and after a standardized PI-QUAL v2 lecture to evaluate accuracy and inter-reader agreement. In Phase II, each hospital submitted clinical prostate MRI scans for centralized quality assurance and quality control; image quality before and after protocol adjustments was compared to determine institutional improvement. PI-QUAL, prostate imaging quality

After the lecture, the same 15 cases were re-evaluated by the participants in a randomized order. A washout period of at least 2 weeks between the first and second image assessments was mandated to minimize recall bias. In this round, all cases were to be assessed strictly according to PI-QUAL v2. The study design is illustrated in Fig. 1.

### Phase II—Quality assurance and control at the institutional level

Beyond teaching the PI-QUAL v2 scoring system, the lecture also aimed to facilitate real-world quality improvement. Each participating institution was encouraged to submit actual prostate MRI cases for post-lecture quality assessment.

Each center was required to submit 15 consecutive prostate MRI exams performed within 1 month before and 1 month after the lecture. Cases included were from patients with suspected prostate cancer but without a known diagnosis. All exams were centrally reviewed using PI-QUAL v2 by the study coordinator, who provided tailored feedback when adjustments were necessary.

The quality assurance (QA) and quality control (QC) processes implemented post-lecture included:

1. Protocol optimization: The coordinator reviewed each site’s imaging protocols. If parameters (e.g., b-values, slice thickness) failed to meet PI-RADS v2.1 standards, adjustments were recommended. Centers already meeting standards retained their original protocols.
2. PI-QUAL v2 adoption and rescanning: Both technologists and radiologists were instructed to use PI-QUAL v2 in routine quality checks. Scans rated as PI-QUAL 1 (i.e., non-diagnostic) required rescanning to meet diagnostic standards.

### Statistical analysis

In Phase I, the primary outcome was the concordance with the expert image-quality reference established by two experienced radiologists using PI-QUAL v2. Concordance was quantified using accuracy and balanced accuracy, the latter defined as the mean of sensitivity and specificity [16]. Because each of the 10 readers assessed the same cases before and after the lecture, differences between phases were evaluated using a logistic mixed-effects model with lecture phase (pre- vs. post-lecture) as a fixed effect and reader and case as random intercepts. Ninety-five percent confidence intervals (CIs) for accuracy and balanced accuracy were estimated by reader- and case-level bootstrap resampling (B = 10,000). The secondary outcome was inter-reader agreement, assessed before and after the lecture, measured by percent agreement (PA) and Gwet’s AC1 coefficient, which is suitable for assessing agreement among multiple raters [17]. Exploratory stratified analyses were performed according to institutional level, participant role, professional level, experience level, knowledge of PI-QUAL, and interval between lecture and reassessment (long > 2 weeks vs. short ≤ 2 weeks).

To facilitate binary analysis, PI-QUAL v2 scores were grouped as score 1 (i.e., non-diagnostic, requiring rescan) and scores 2–3 (i.e., acceptable/adequate diagnostic quality). Before the lecture, participants answered whether an image required rescanning: ‘rescan’ indicated poor quality, whereas ‘no need to rescan’ indicated acceptable/adequate image quality. After the lecture, participants assigned PI-QUAL v2 scores, which were dichotomized using the same threshold. Inter-reader agreement was also evaluated for each PI-QUAL criterion using binary scoring. Gwet’s AC1 interpretation was as follows: < 0.00: Poor; 0.00–0.20: Slight; 0.21–0.40: Fair; 0.41–0.60: Moderate; 0.61–0.80: Substantial; 0.81–1.00: Almost perfect.

In Phase II, the proportion of diagnostic-quality scans (PI-QUAL v2 > 1) before and after the lecture was calculated. Statistical analyses were conducted using R version 4.2.2 (R Foundation for Statistical Computing, Vienna, Austria), with a two-sided *p*-value < 0.05 considered statistically significant.

## Results

### Participant characteristics

A total of five radiologists and five technologists from five different hospitals took part in this study. Among them, four hospitals were tertiary centers and one was a secondary hospital. Of the ten participants, two radiologists held junior-level titles, three radiologists and four technologists were at the intermediate level, and one technologist was at the senior level. Regarding prostate MRI experience, one radiologist was a beginner, four radiologists and one technologist had intermediate experience, and four technologists were considered experts. Five participants reported having never heard of PI-QUAL, while the remaining five had basic knowledge of PI-QUAL. Half of the participants completed the second round of image-quality evaluation within 2 weeks after the lecture, and the other half completed it after more than 2 weeks (Table 1).

**Table 1:** The characteristics of the evaluators who participated in this study

Evaluator	Hospital	Hospital level	Role	Professional title	Experience in prostate MRI	Knowledge of PI-QUAL	Interval with lecture*
1	FHPH	Secondary	Radiologist	Intermediate	Intermediate	Basic	Long
2			Technologist	Intermediate	Expert	Basic	Short
3	HZCH	Tertiary	Radiologist	Junior	Intermediate	None	Long
4			Technologist	Senior	Expert	None	Short
5	ZJCH	Tertiary	Radiologist	Intermediate	Intermediate	Basic	Short
6			Technologist	Intermediate	Intermediate	Basic	Long
7	SXPH	Tertiary	Radiologist	Intermediate	Beginner	None	Short
8			Technologist	Intermediate	Expert	None	Long
9	PUMCH	Tertiary	Radiologist	Junior	Intermediate	None	Long
10			Technologist	Intermediate	Expert	Basic	Short

*FHPH* Fenghua People’s Hospital, *HZCH* Huzhou Central Hospital, *PUMCH* Peking Union Medical College Hospital, *SXPH* Shaoxing People’s Hospital, *ZJCH* Zhejiang Cancer Hospital, *PI-QUAL* prostate imaging quality

* The interval between the lecture and reassessment was categorized as long (> 2 weeks) or short (≤ 2 weeks)

### Accuracy of image-quality evaluation before and after the lecture

**Fig. 2:**
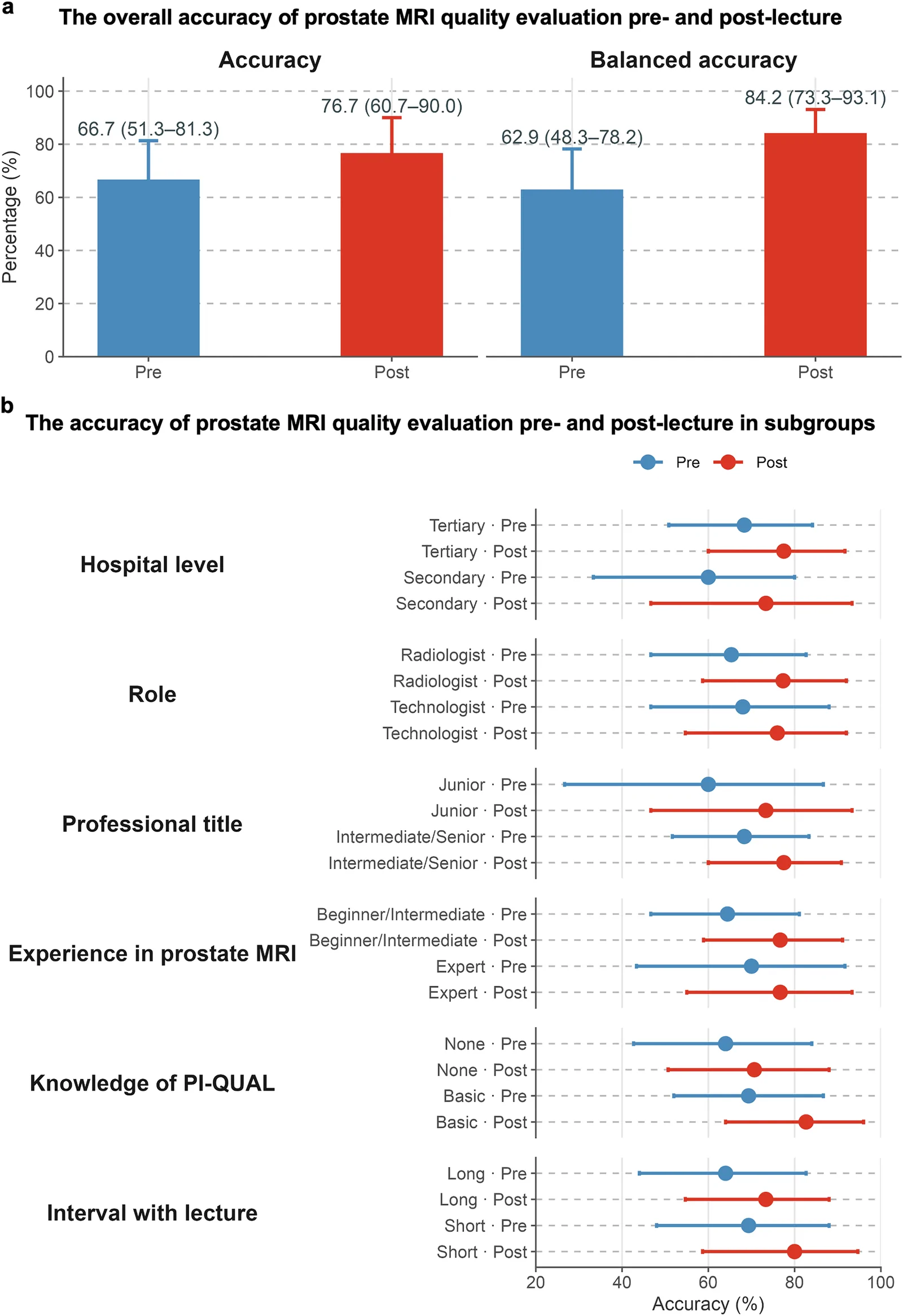
Concordance of prostate MRI quality evaluation compared with the reference pre- and post-lecture. **a** Overall accuracy and balanced accuracy pre- and post-lecture. **b** Accuracy in subgroups. Overall accuracy increased from 66.7% to 76.7%, and balanced accuracy increased from 62.9% to 84.2%. Subgroup accuracy improved from 60.0–70.0% to 70.7–82.7%. PI-QUAL, prostate imaging quality

Among the 15 cases selected for analysis, PI-QUAL v2 scores were distributed as follows: 3 cases scored 1, 7 scored 2, and 5 scored 3. A total of 150 pre-lecture and 150 post-lecture reader assessments were collected. Overall accuracy increased from 66.7% to 76.7%, and balanced accuracy increased from 62.9% to 84.2%; correct assessments were significantly more likely after the lecture than before it in the mixed-effects model (*p* = 0.038; Table 2 and Fig. 2).

**Table 2:** The accuracy and balanced accuracy of prostate MRI quality evaluation compared with the reference pre- and post-lecture

Categories	Subgroup analysis	Number of evaluators	Concordance with reference (95% CI, %)			
			Pre accuracy	Post accuracy	Pre balanced accuracy	Post balanced accuracy
Overall	-	10	66.7 (51.3–81.3)	76.7 (60.7–90.0)	62.9 (48.3–78.2)	84.2 (73.3–93.1)
Hospital level	Tertiary	8	68.3 (50.8–84.2)	77.5 (60.0–91.7)	61.5 (43.3–80.1)	85.9 (76.0–94.3)
	Secondary	2	60.0 (33.3–80.0)	73.3 (46.7–93.3)	68.8 (44.2–88.5)	77.1 (50.0–96.2)
Role	Radiologist	5	65.3 (46.7–82.7)	77.3 (58.7–92.0)	60.8 (40.0–81.0)	83.3 (67.2–94.6)
	Technologist	5	68.0 (46.7–88.0)	76.0 (54.7–92.0)	65.0 (46.4–85.6)	85.0 (72.7–95.0)
Professional title	Junior	2	60.0 (26.7–86.7)	73.3 (46.7–93.3)	50.0 (20.0–91.1)	83.3 (69.2–95.8)
	Intermediate/Senior	8	68.3 (51.7–83.3)	77.5 (60.0–90.8)	66.1 (52.7–80.4)	84.4 (71.9–93.8)
Experience in prostate MRI	Beginner/Intermediate	6	64.4 (46.7–81.1)	76.7 (58.9–91.1)	59.0 (39.2–78.2)	83.3 (69.9–93.5)
	Expert	4	70.0 (43.3–91.7)	76.7 (55.0–93.3)	68.8 (49.7–91.3)	85.4 (72.2–96.2)
Knowledge of PI-QUAL	None	5	64.0 (42.7–84.0)	70.7 (50.7–88.0)	65.0 (41.4–87.3)	81.7 (70.8–91.7)
	Basic	5	69.3 (52.0–86.7)	82.7 (64.0–96.0)	60.8 (45.7–78.3)	86.7 (70.7–97.3)
Interval with lecture	Long	5	64.0 (44.0–82.7)	73.3 (54.7–88.0)	60.0 (35.7–85.0)	80.8 (66.5–92.0)
	Short	5	69.3 (48.0–88.0)	80.0 (58.7–94.7)	65.8 (49.3–83.6)	87.5 (75.0–96.9)

Values are percentages; parentheses denote 95% CIs. Balanced accuracy is the mean of sensitivity and specificity. CIs were estimated by reader- and case-level percentile bootstrap resampling (B = 10,000)

*PI-QUAL* prostate imaging quality, *CI* confidence interval

Subgroup analyses showed higher post-lecture accuracy in all subgroups, with pre-lecture values ranging from 60.0% to 70.0% and post-lecture values ranging from 70.7% to 82.7%. Balanced accuracy was also higher after the lecture in all subgroups (pre-lecture, 50.0–68.8%; post-lecture, 77.1–87.5%). The largest numerical gains were observed among participants who completed the second-round evaluation within 2 weeks after the lecture and among those with basic knowledge of PI-QUAL (Table 2 and Fig. 2).

### Inter-observer agreement of imaging quality before and after the lecture

**Fig. 3:**
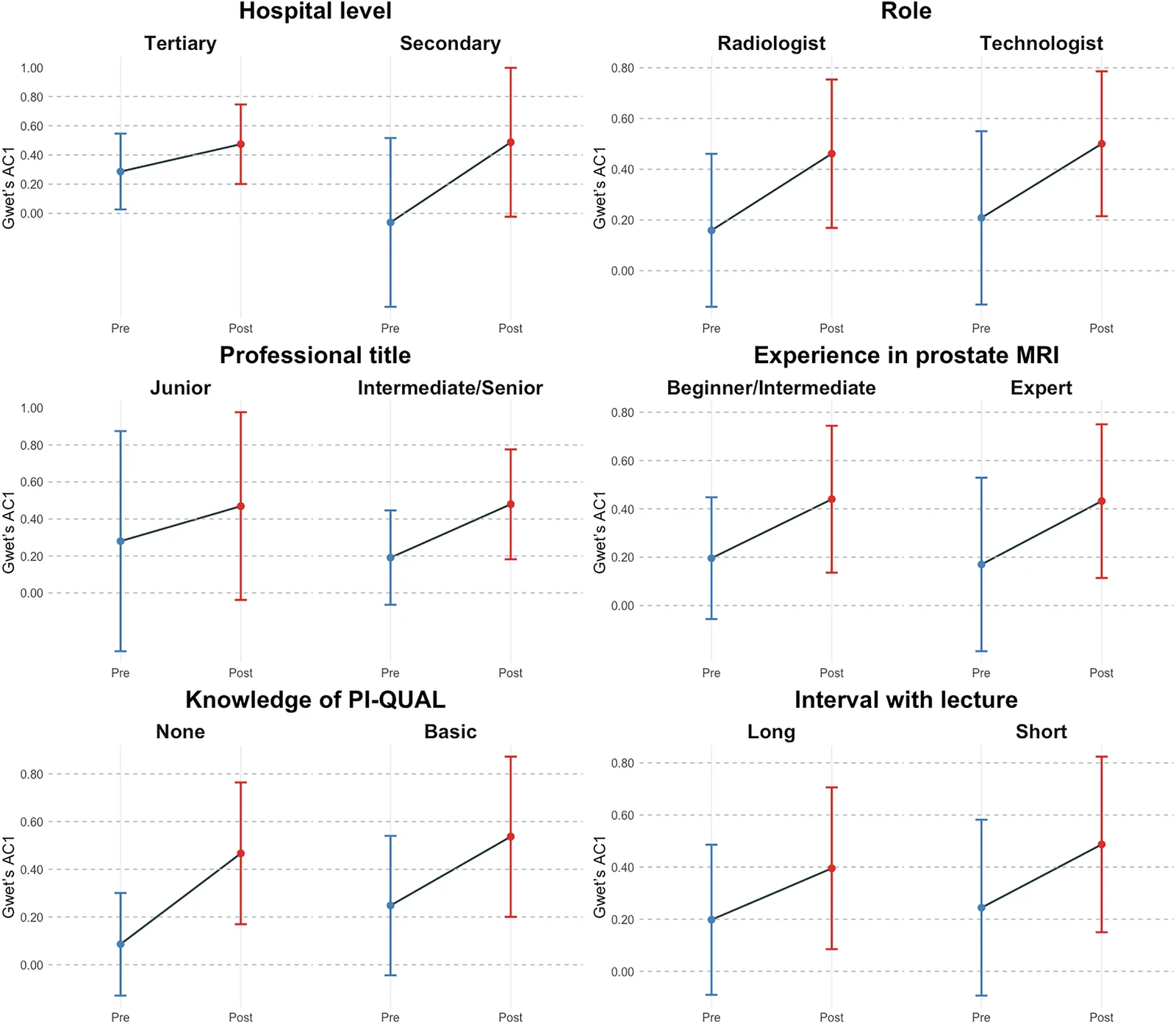
The inter-reader agreement for prostate MRI quality evaluation pre- and post-lecture. Subgroup analyses showed improvements in inter-reader agreement across participants stratified by hospital level, role in prostate MRI, professional title, prostate MRI experience, PI-QUAL knowledge, and interval from the lecture. Pre-lecture agreement ranged from poor to fair (AC1: −0.062 to 0.286), while post-lecture agreement ranged from fair to moderate across all groups (AC1: 0.395–0.537). PI-QUAL, prostate imaging quality

Before the lecture, the overall PA among all participants was 57.6%, with a Gwet’s AC1 coefficient of 0.214, indicating fair agreement. After the lecture, agreement improved, with PA increasing to 72.6% and AC1 rising to 0.466 (Table 3). Subgroup analyses revealed that pre-lecture inter-reader agreement ranged from poor to fair (AC1: −0.062 to 0.286), while post-lecture agreement ranged from fair to moderate (AC1: 0.395 to 0.537) across all groups (Fig. 3). For the inter-reader agreement of individual PI-QUAL v2 criteria after the lecture, the highest agreement was observed for the DCE sequence, with AC1 values ranging from 0.781 to 0.885, followed by T2-weighted imaging (T2WI) (AC1: 0.456–0.634). The lowest agreement was noted for DWI, especially in evaluating the signal-to-noise ratio (SNR), which had an AC1 of −0.022 (Table S3 and Fig. S1).

**Table 3:** The inter-reader agreement for prostate MRI quality evaluation pre- and post-lecture

Categories	Subgroup analysis	Number of evaluators	PA (%)		Gwet’s AC1	
			Pre	Post	Pre	Post
All	-	10	57.6	72.6	0.214 (0.000–0.429)	0.466 (0.195–0.736)
Hospital level	Tertiary	8	59.5	73.1	0.286 (0.027–0.546)	0.474 (0.201–0.746)
	Secondary	2	46.7	73.3	−0.062 (−0.64–0.516)	0.487 (−0.023–0.997)
Role	Radiologist	5	54.7	72.0	0.159 (−0.142–0.461)	0.462 (0.169–0.754)
	Technologist	5	57.3	74.7	0.209 (−0.133–0.550)	0.501 (0.215–0.786)
Professional title	Junior	2	60.0	73.3	0.280 (−0.315–0.875)	0.469 (−0.038–0.977)
	Intermediate/Senior	8	56.7	73.1	0.191 (−0.064–0.446)	0.479 (0.182–0.776)
Experience in prostate MRI	Beginner/Intermediate	6	56.4	71.1	0.196 (−0.056–0.448)	0.440 (0.136–0.744)
	Expert	4	55.6	71.1	0.170 (−0.189–0.529)	0.432 (0.114–0.750)
Knowledge of PI-QUAL	None	5	53.3	73.3	0.086 (−0.129–0.301)	0.467 (0.170–0.764)
	Basic	5	56.0	74.7	0.248 (−0.044–0.540)	0.537 (0.201–0.872)
Interval with lecture	Long	5	57.3	69.3	0.198 (−0.090–0.486)	0.395 (0.085–0.706)
	Short	5	58.7	73.3	0.244 (−0.093–0.582)	0.487 (0.15–0.824)

*PI-QUAL* prostate imaging quality, *PA* percent agreement

### Institution-level image-quality improvement after the lecture

**Fig. 4:**
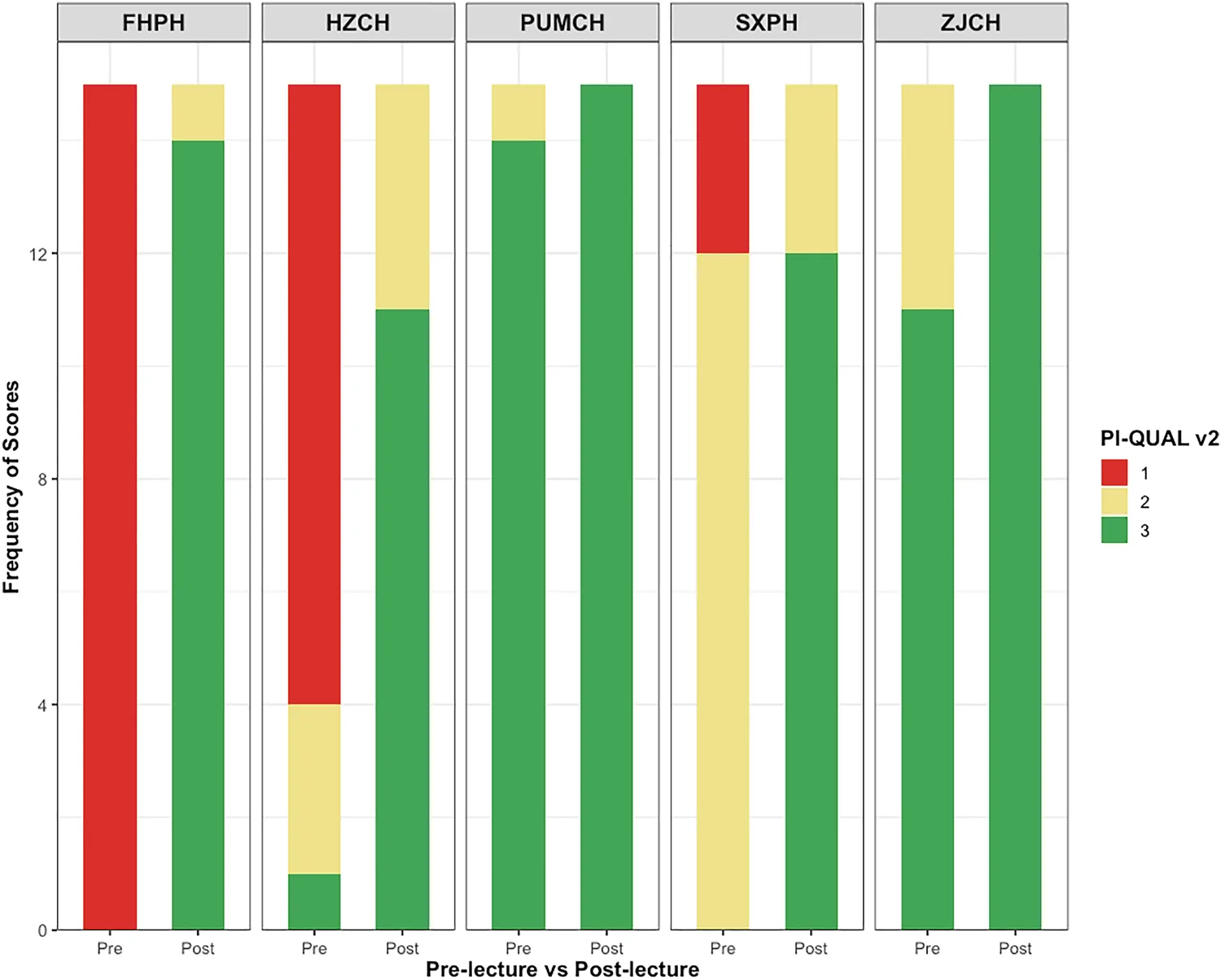
The hospital-level image-quality improvements after the lecture (*n* (%)). Prior to the lecture, the number of cases with PI-QUAL v2 scores > 1 was 15/15 at PUMCH and ZJCH, 12/15 at SXPH, 4/15 at HZCH, and 0/15 at FHPH. After quality assurance and improvement measures, all centers achieved PI-QUAL v2 scores > 1 in 15/15 of the newly scanned cases. FHPH, Fenghua People’s Hospital; HZCH, Huzhou Central Hospital; PUMCH, Peking Union Medical College Hospital; SXPH, Shaoxing People’s Hospital; ZJCH, Zhejiang Cancer Hospital; PI-QUAL, prostate imaging quality

Baseline image quality varied considerably across institutions. Prior to the lecture, the proportion of cases with a PI-QUAL v2 score > 1 was 100% (15/15) at PUMCH and ZJCH, 80% (12/15) at SXPH, 26.7% (4/15) at HZCH, and 0.0% (0/15) at FHPH. Three centers did not comply with the PI-QUAL v2 minimum technical requirements and were advised to revise their scanning protocols (Table S4). Specifically, two centers demonstrated deviations in axial T2WI slice thickness and the absence of DWI with b ≤ 1000 s/mm² required for apparent diffusion coefficient map calculation, while three centers showed prolonged DCE temporal resolution (> 15 s). One center required optimization limited to the DCE sequence, whereas the other two centers required parameter adjustments across multiple sequences (T2WI, DWI, and DCE). Following the implementation of quality assurance and improvement measures, all centers achieved PI-QUAL v2 scores > 1 in 100% of the newly scanned 15 cases (Fig. 4).

## Discussion

This pilot study suggests that an educational intervention based on PI-QUAL v2 can improve both the accuracy of prostate MRI image-quality evaluation among individual readers and the actual image quality at the institutional level. Following the PI-QUAL v2 lecture, evaluators showed improved ability to assess image quality objectively and consistently, regardless of their baseline experience or professional background. Importantly, the educational session also encouraged institutional engagement in QA/QC practices. Notable improvements in adherence to technical standards and image quality scores were observed after the intervention.

Our findings indicate that the PI-QUAL v2 lecture improved image-quality assessment across all evaluator subgroups, regardless of background. The first study evaluating the impact of a prostate MRI quality training lecture combined with a hands-on workshop was published in 2023 [14]. The study included 16 participants (radiologists, urologists, physicists, and computer scientists) with varying levels of experience in reviewing diagnostic prostate MRI. A significant improvement in AUC was observed for the image-quality evaluation, which increased from 0.59 at baseline to 0.96 post-intervention. Another online-based PI-QUAL lecture involving 18 radiology trainees also reported an increase in AUC from 0.82 to 1.00 [18]. Stratified analysis in that study showed the greatest benefit among less experienced trainees, with AUC improvements of 0.48 in second-year residents, 0.32 in fourth-year residents, and 0.1 in abdominal imaging fellows. With the release of PI-QUAL v2, Curia et al reported the first results of the PI-QUAL v2 educational program for technologists, demonstrating that the training module enhanced the ability of both MRI technology students and board-certified MRI technologists to assess prostate MRI image quality [19]. Compared with previous published studies, our pilot study enrolled pairs of radiologists and technologists from the same hospital, as they were actively involved in prostate MRI practice. Subgroup analysis demonstrated improvement in image-quality assessment regardless of participants’ role in prostate MRI or their professional background. Radiologists and technologists directly engaged in daily prostate MRI practice benefited from the structured lecture, enabling them to score image quality more accurately. Notably, participants who completed the reassessment within 2 weeks after the lecture showed larger numerical gains, possibly reflecting short-term reinforcement of newly acquired knowledge and partial knowledge decay over longer intervals. This observation highlights the potential benefit of integrating PI-QUAL v2 into routine clinical practice to maintain consistent scoring proficiency.

The inter-reader agreement in our study remained moderate. Prior studies have shown varying levels of agreement for PI-QUAL v2. Orman et al [20] reported high inter-reader agreement among five readers for 274 cases, with weighted Gwet’s AC1 values ranging from 0.821 to 0.897, which were higher than those for PI-QUAL v1 (0.659 to 0.855). In contrast, Ponsiglione et al [12] reported a moderate inter-reader agreement (Gwet’s AC1 = 0.55) across six readers, with higher agreement among expert readers (AC1 = 0.66) compared to beginners and basic ones (AC1 = 0.45–0.58). Similarly, Lee et al [21] reported a Cohen’s κ of 0.54 for mpMRI assessments using PI-QUAL v2. The relatively small sample size in our study, along with the predominance of intermediate-quality images (PI-QUAL v2 score: 2), may have posed challenges for participants in evaluating image quality, potentially contributing to the lower AC1 values in our study. Nevertheless, we observed a notable improvement in inter-reader agreement after the lecture, increasing from 0.214 to 0.466. This suggests that PI-QUAL v2 can facilitate more consistent evaluations than subjective assessment alone. Notably, agreement was highest for the DCE sequence, followed by T2WI, and lowest for DWI. The relatively low agreement for DWI quality evaluation observed in our study, particularly for SNR assessment, is consistent with previous studies involving multi-scanner image-quality evaluation and may represent an important contributor to inter-reader variability in the final PI-QUAL v2 classification [22, 23]. The relatively low agreement in DWI SNR assessment likely reflects the subjective nature of the current evaluation criteria. Future PI-QUAL training and guideline updates may benefit from incorporating quantitative SNR metrics and objective reference standards [24], which might help improve reproducibility and reduce inter-reader variability.

The educational lecture, followed by QA/QC procedures, contributed to institutional-level improvements in image quality. To promote real-world application of PI-QUAL, all participating centers were required to submit 15 prostate MRI cases before and after the intervention. Two centers had already adopted standardized PI-RADS protocols, while three required adjustments due to noncompliance with technical standards. After optimization, all post-intervention scans achieved PI-QUAL v2 scores > 1, indicating diagnostic-quality imaging. High-quality imaging is essential for the accurate diagnosis of prostate cancer. Shin et al [25] found that PI-QUAL v2 scores < 2 were associated with reduced sensitivity and negative predictive value for detecting clinically significant prostate cancer. Similarly, a quality improvement initiative [26] improved the proportion of PI-QUAL v1 score ≥ 4 exams from 67% to 84% (*p* < 0.001), with positive predictive value increasing from 65% to 78% (*p* = 0.03). Compared with previous studies, the follow-up period in our study was relatively short. Further long-term follow-up is warranted to evaluate the sustainability of the observed improvements and the potential benefits of PI-QUAL implementation on image acquisition and diagnostic performance.

The American College of Radiology Learning Network has demonstrated success in improving image quality across institutions through shared data and targeted interventions [27, 28]. Its Prostate MR Image Quality Improvement Collaborative set a goal of achieving PI-QUAL v1 scores ≥ 4 in at least 85% of examinations at each participating site [29]. In their initial audit of 2380 examinations, the mean weekly rate of exams meeting image quality standards increased from 67% at baseline to 87% post-intervention. Common interventions included MR protocol optimization, development and implementation of patient preparation instructions, personnel training, and development of an auditing process mechanism. At the University of Rochester Medical Center, efforts to enhance MRI quality included improving patient preparation, fostering radiologist-technologist collaboration, and adopting standardized image quality scoring [30]. These measures increased the percentage of exams with PI-QUAL score ≥ 4 from 90% to 97%, and the proportion meeting DWI criteria from 77% to 83%. Achieving and sustaining high-quality prostate MRI at the institutional level requires ongoing commitment and structured quality control initiatives.

This study has several limitations. First, the number of cases evaluated was limited, and most were assigned PI-QUAL v2 scores of 2, which may have posed challenges for readers to reach consensus, thereby affecting inter-reader agreement and generalizability. Larger studies including more cases with a broader distribution of image quality levels are needed to provide a more robust evaluation of PI-QUAL v2 reproducibility and identify strategies to improve inter-reader agreement. Prospective studies involving additional readers and cases are currently underway to further validate these findings and explore approaches for improving PI-QUAL v2 implementation. Second, due to time constraints, no hands-on workshop was conducted, which could have further strengthened participants’ understanding. Finally, the QA/QC processes were relatively short and dependent on voluntary participation. Long-term monitoring and periodic re-auditing will be necessary to evaluate the sustainability of the quality improvement strategy.

In conclusion, our pilot study indicates that a dedicated PI-QUAL lecture can improve the accuracy of prostate MRI image-quality evaluation and reduce inter-reader variability among radiologists and technologists with diverse backgrounds. Moreover, the online lecture, combined with tailored QA/QC feedback, effectively promotes institutional-level improvements in imaging practice. Our findings emphasize the importance of structured image quality education in standardizing prostate MRI across institutions and enhancing evaluation consistency. Future work should consider incorporating hands-on workshops and continuous auditing mechanisms to further optimize and sustain imaging quality. Establishing a nationwide quality control network based on PI-QUAL could be a promising step toward improving prostate cancer diagnosis and care.

## Supplementary information

ELECTRONIC SUPPLEMENTARY MATERIAL

## Data Availability

The datasets used and/or analyzed during the current study are available from the corresponding author on reasonable request.

## Abbreviations

DCE: Dynamic contrast-enhanced
DWI: Diffusion-weighted imaging
PA: Percent agreement
PI-QUAL: Prostate imaging quality
PI-RADS: Prostate Imaging Reporting and Data System
QA: Quality assurance
QC: Quality control
T2WI: T2-weighted imaging
