# Supplementary material for: Improving prostate MRI quality assessment across diverse evaluators and institutional practice through a PI-QUAL v2 lecture: a pilot study
Source: Insights Imaging. 2026 Oct 29;17:269. doi: 10.1186/s13244-026-02450-x (PMC13719960; doi:10.1186/s13244-026-02450-x)
Supplement: Supplementary file 1 [file 13244_2026_2450_MOESM1_ESM.pdf]

Improving Prostate MRI Quality Assessment Across Diverse Evaluators and Institutional Practice Through a PI-QUAL v2 Lecture: A Pilot Study

ELECTRONIC SUPPLEMENTARY MATERIAL

Table S1. The characteristics of the cases selected for evaluation

Number	Age	Hospital	Vendor	Scanner	Field strength	T2WI score	DWI score	DCE score	PI-QUAL v2
1	81	PUMCH	GE	DISCOVERY 750	3 T	2	2	+	1
2	63	PUMCH	Philips	Ingenia Elition X	3 T	4	4	+	3
3	85	PUMCH	Philips	Ingenia Elition X	3 T	4	4	+	3
4	73	PUMCH	GE	DISCOVERY 750	3 T	1	1	+	1
5	63	PUMCH	GE	DISCOVERY 750	3 T	4	2	+	2
6	62	PUMCH	GE	DISCOVERY 750	3 T	2	2	+	1
7	75	PUMCH	GE	DISCOVERY 750	3 T	4	2	+	2
8	65	PUMCH	GE	DISCOVERY 750	3 T	4	2	+	2
9	71	ZJCH	Philips	Ingenia CX	3 T	2	4	+	2
10	67	ZJCH	Philips	Ingenia CX	3 T	2	4	+	2
11	67	ZJCH	Philips	Ingenia CX	3 T	2	4	+	2
12	47	ZJCH	Philips	Ingenia CX	3 T	4	4	+	3
13	76	ZJCH	Philips	Ingenia CX	3 T	4	4	+	3
14	54	ZJCH	Philips	Ingenia CX	3 T	4	4	+	3
15	60	ZJCH	Philips	Ingenia CX	3 T	2	4	+	2

Note—PUMCH, Peking Union Medical College Hospital; ZJCH, Zhejiang Cancer Hospital; PI-QUAL, Prostate Imaging Quality; T2WI, T2-weighted imaging; DWI, diffusion-weighted imaging; DCE, dynamic contrast-enhanced imaging.

Table S2: Questionnaire for pre-lecture MRI evaluation

**Name**		
**Hospital**		
**Role in prostate MRI**		£ Radiologist £ Technologist
**Professional title**		£ Junior: Resident Radiologist/Junior Radiology Technologist<br>£ Intermediate: Attending Radiologist/ Senior Radiology Technologist<br>£ Senior： Associate Chief or Chief Radiologist/Associate Chief or Chief Radiology Technologist
**Experience in prostate MRI (months/years)**		
**Number of prostate MRIs reported/scanned per year**		
**Have you ever heard of PI-QUAL?**		£ Yes £ No
**Do you know the scoring criteria of PI-QUAL?**		£ Yes (full) £ Yes (basic) £ None
**Does your institution implant PI-QUAL in image quality evaluation?**		£ Yes £ No
**Image quality assessment**<br>*Please assess the image quality for each sequence and state the reason of your evaluation*		
**Number**		
**T2WI**	**Image quality**	£ Adequate £ Sub-optimal £ Inadequate
	**Reason**	
**DWI/ADC**	**Image quality**	£ Adequate £ Sub-optimal £ Inadequate
	**Reason**	
**DCE**	**Image quality**	£ Adequate £ Sub-optimal £ Inadequate

	**Reason**	
**Would you feel confident in diagnosing or excluding prostate cancer based on these images?**		£ Yes £ No
**Would you repeat the scan?**		£ Yes £ No

Note—PI-QUAL, Prostate Imaging Quality; T2WI, T2-weighted imaging; DWI, diffusion-weighted imaging; ADC, apparent diffusion coefficient; DCE, dynamic contrast-enhanced imaging.

Table S3. The inter-reader agreement of the individual PI-QUAL v2 criteria

	Gwet's AC1
**PI-QUAL v2 (>1 vs =1)**	0.466 (0.195–0.736)
**T2WI (>2 vs ≤2)**	0.648 (0.423–0.874)
Axial: adequate SNR in all parts of the images	0.580 (0.305–0.856)
Axial: ability to clearly delineate relevant structures in the prostate	0.634 (0.349–0.919)
Axial: absence of significant artefacts in the prostatic region	0.626 (0.354–0.899)
Sagittal OR coronal: adequate SNR and image resolution AND absence of significant artefacts	0.456 (0.191–0.721)
**DWI (>2 vs ≤2)**	0.377 (0.141–0.614)
Adequate contrast and SNR on high b value images	-0.022 (-0.065–0.022)
Adequate range of contrast to differentiate TZ/BPH from PZ on the ADC maps	0.419 (0.134–0.704)
Absence of significant artefacts in the prostatic region	0.532 (0.286–0.779)
Anatomical matching of the ADC map / high b value sequence to the axial T2-WI	0.632 (0.390–0.874)
**DCE**	0.678 (0.472–0.884)
Absence of significant artefacts in the prostatic region and appropriate bolus enhancement	0.781 (0.616–0.946)
Ability to identify anatomical structures (e.g. capsular vessels or pudendal artery)	0.885 (0.802–0.967)

Note—PI-QUAL, Prostate Imaging Quality; T2WI, T2-weighted imaging; DWI, diffusion-weighted imaging; ADC, apparent diffusion coefficient; DCE, dynamic contrast-enhanced imaging.

Table S4. Prostate MRI acquisition parameters before and after protocol review and optimization across participating centers

Hospital	Vendor	Scanner	Field strength	PI-QUAL v2 compliant		T2WI		DWI			DCE	
						ST/gap (mm)	Additional plane	ST/gap (mm)	b values (s/mm²)	Fat saturation	ST/gap (mm)	Temporal resolution (s)
**FHPH**	Siemens	Avanto	1.5 T	×	Before	**3.5/0.7**	Sag, Cor	**3.5/0.7**	**0, 1400**	With	2/0	**24**
					After	3/0	-	3/0	0, 800, 1400	-	3/0	15
**HZCH**	GE	DISCOVERY 750	3 T	×	Before	**4/1**	Cor	**4/1**	**0, 1800**	With	3/0	**28**
					After	3/0	Sag, Cor	3/0	50, 800, 1400*	-	-	15
**PUMCH**	GE	DISCOVERY 750	3 T	√		3/0	Sag, Cor	3/0	0, 100, 500, 800, 1000, 1500, 2000	With	3/0	14
	GE	DISCOVERY 750w	3 T	√		3/0	Sag, Cor	3/0	0, 100, 800, 1500	With	3/0	14
	Philips	Ingenia Elition X	3 T	√		3/0	Sag, Cor	4/0	0, 100, 800, 1000, 1500, 2000	With	3/0	14
**SXPH**	Siemens	MAGNETOM Vida	3 T	×	Before	3/0	Sag, Cor	3/0	50, 800, 1500	With	3/0	**22**
					After	-	-	-	-	-	-	15
**ZJCH**	Philips	Ingenia CX	3 T	√		3/0	Sag, Cor	3/0	50, 800, 1200, 1500	With	3/0	15

GE	SIGNA Premier	3 T	√	3/0	Sag, Cor	3/0	50, 800, 1200, 1500	With	3/0	15

Note—FHPH, Fenghua People’s Hospital; HZCH, Huzhou Central Hospital; PUMCH, Peking Union Medical College Hospital; SXPH, Shaoxing People's Hospital; ZJCH, Zhejiang Cancer Hospital; PI-QUAL, Prostate Imaging Quality; T2WI, T2-weighted imaging; DWI, diffusion-weighted imaging; DCE, dynamic contrast-enhanced imaging; ST, slice thickness.

Parameters that did not meet PI-QUAL v2 minimum technical requirements during pre-optimization central review are highlighted in bold.

* High b-value images were synthetically generated.

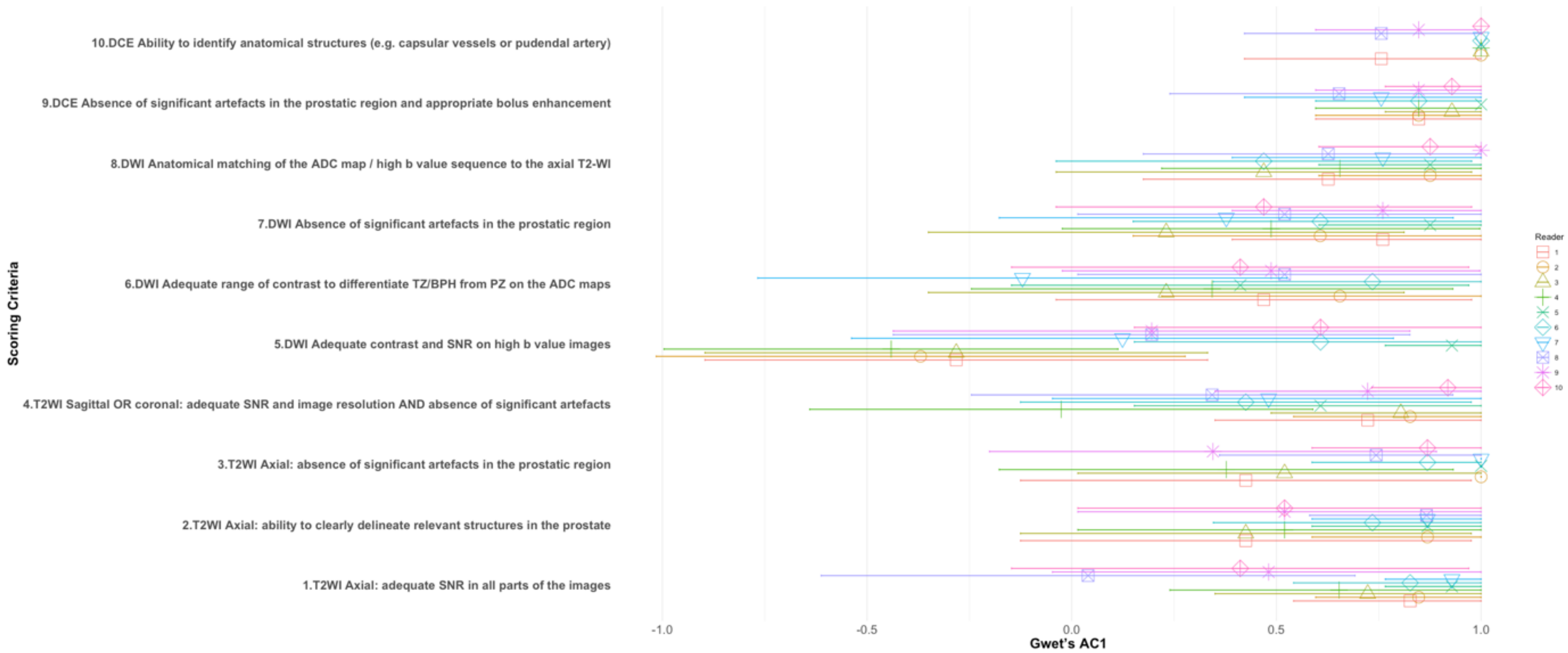

Figure S1. The plot of Gwet's AC1 value for the detailed scoring criteria of the independent evaluator

Note—T2WI, T2-weighted imaging; DWI, diffusion-weighted imaging; ADC, apparent diffusion coefficient; DCE, dynamic contrast-enhanced imaging.
